# Supplementary figures and images for: Immune cell single-cell RNA sequencing analyses link an age-associated T cell subset to symptomatic benign prostatic hyperplasia
Source: Front Immunol. 2025 Jul 7;16:1585446. doi: 10.3389/fimmu.2025.1585446 (PMC12278823; doi:10.3389/fimmu.2025.1585446)

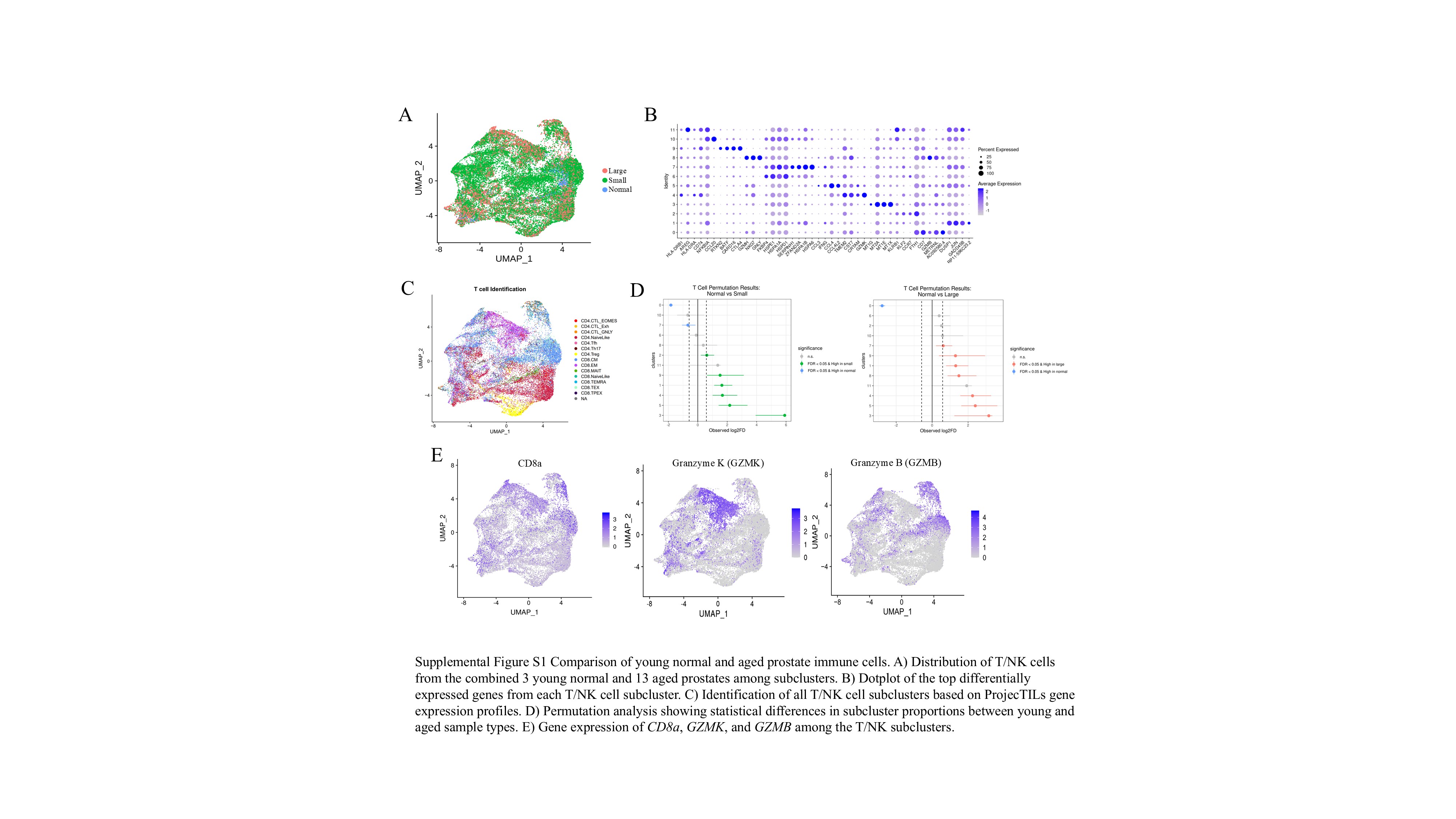

Supplement: Supplementary file 1 [file Image1.jpeg]

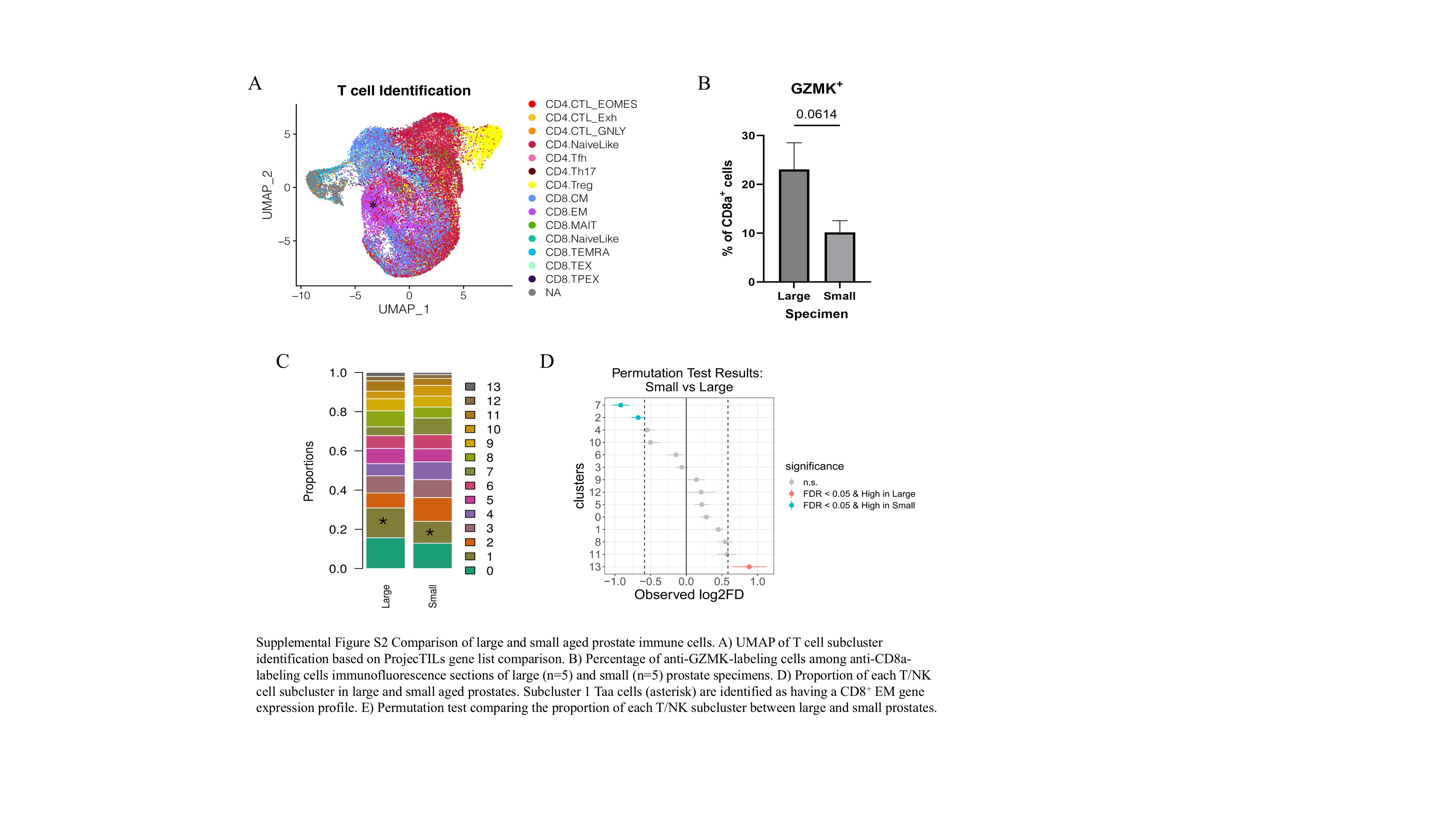

Supplement: Supplementary file 2 [file Image2.jpeg]

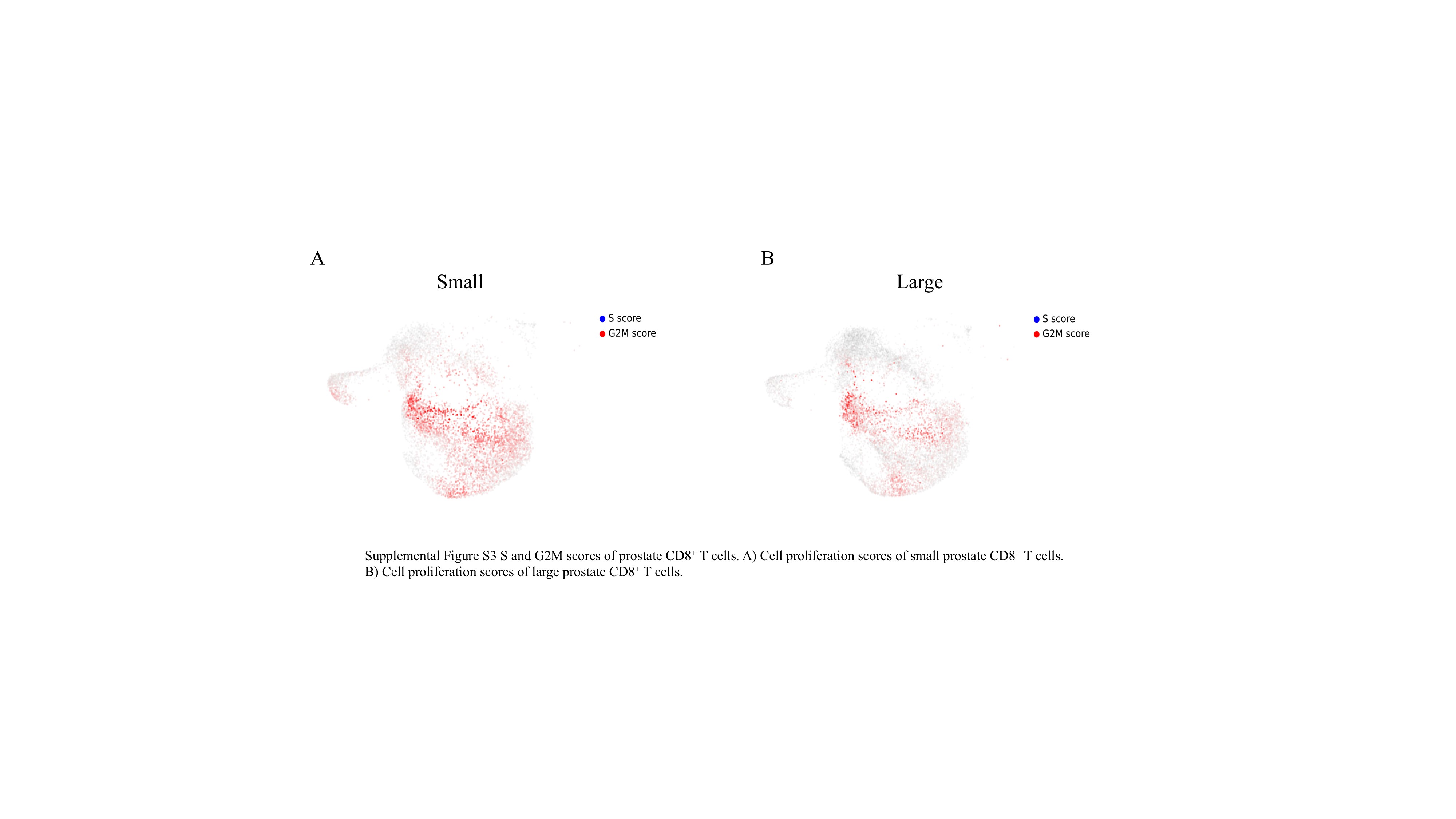

Supplement: Supplementary file 3 [file Image3.jpeg]

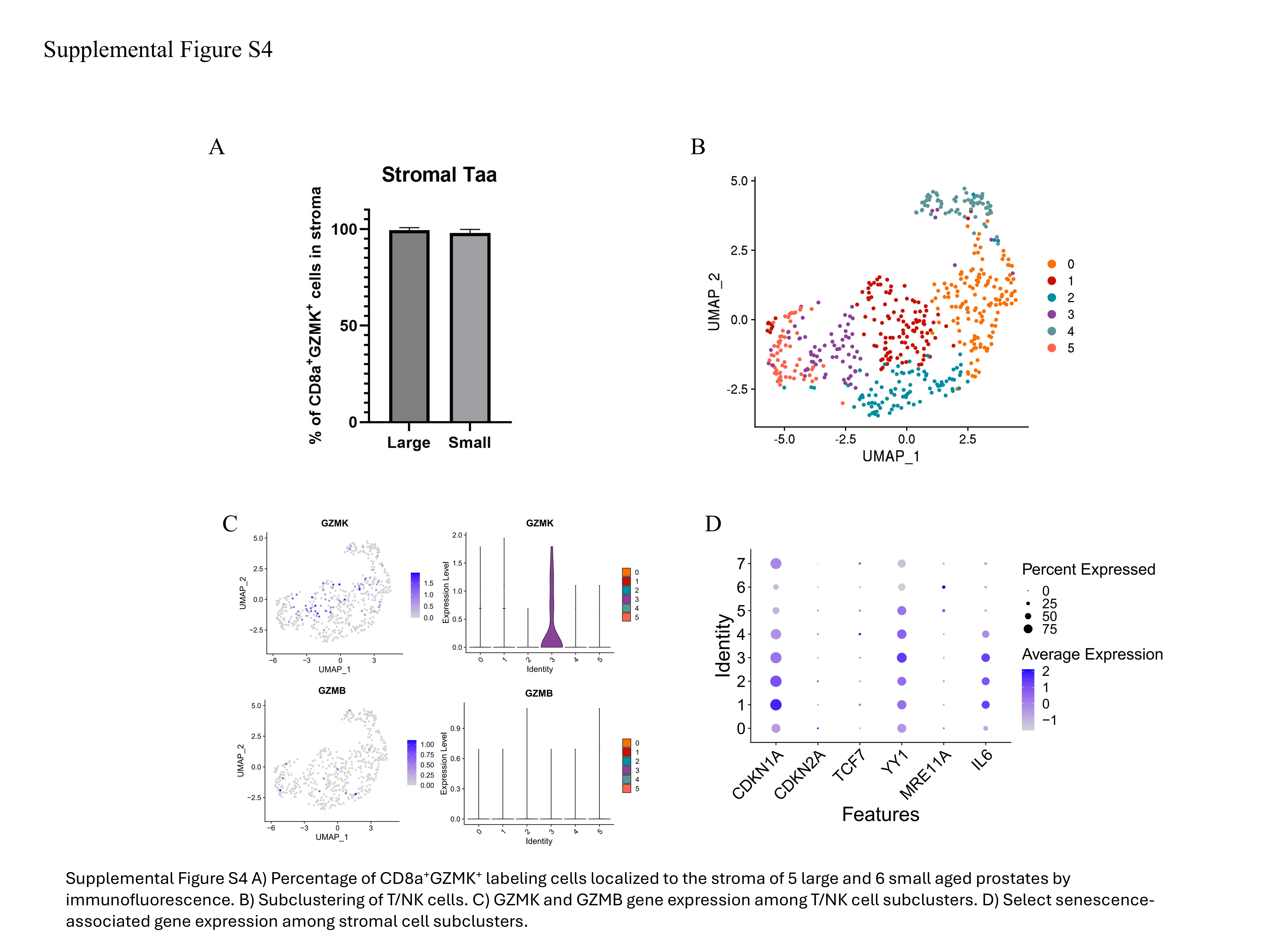

Supplement: Supplementary file 4 [file Image4.jpeg]
